# The biological function and clinical significance of STIL in osteosarcoma

**DOI:** 10.1186/s12935-021-01922-y

**Published:** 2021-04-15

**Authors:** Shu-fan Ji, Sheng-Lian Wen, Yu Sun, Pi-wei Huang, Hao Wu, Mao-lin He

**Affiliations:** 1 Division of Spinal Surgery, The First Affiliated Hospital of Guangxi Medical University, Shuangyong Road 6, Nanning, 530021 Guangxi Zhuang Autonomous Region People’s Republic of China; 2 Department of Radiology, The First Affiliated Hospital, Guangxi Medical University, Shuangyong Road 6, Guangxi Zhuang Autonomous Region 530021 Nanning, People’s Republic of China; 3 Guangxi Collaborative Innovation Center for Biomedicine, Guangxi Medical University, Nanning, 530021 Guangxi Zhuang Autonomous Region People’s Republic of China; 4 Guangxi-ASEAN Collaborative Innovation Center for Major Disease Prevention and Treatment, Guangxi Medical University, Nanning, 530021 Guangxi Zhuang Autonomous Region People’s Republic of China

**Keywords:** STIL, Osteosarcoma, Proliferation, Invasion, Biomarkers

## Abstract

**Background:**

SCL/TAL1 interrupting locus (STIL) is associated with the progression of several tumors; however, the biological role of STIL in osteosarcoma remains poorly understood.

**Methods:**

In this study, the clinical significance of STIL in osteosarcoma was analyzed by gene chip data recorded in public databases. STIL expression was silenced in osteosarcoma cell lines to observe the effects on proliferation, apoptosis, invasion, and migration. Differentially expressed genes (DEGs) in the osteosarcoma chip were analyzed using The Limma package, and STIL co-expressed genes were obtained via the Pearson correlation coefficient. The potential molecular mechanism of STIL in osteosarcoma was further explored by Gene Ontology (GO) and Kyoto Encyclopedia of Genes and Genomes (KEGG) pathways.

**Results:**

Osteosarcoma was associated with higher STIL expression compared to the control samples, and the standardized mean difference (SMD) was 1.52. STIL also had a good ability to distinguish osteosarcoma from non-osteosarcoma samples [area under the curve (AUC) = 0.96]. After silencing STIL, osteosarcoma cell proliferation decreased, apoptosis increased, and the migratory and invasion ability decreased. A total of 294 STIL differentially co-expressed genes were screened, and a bioinformatics analysis found that differentially co-expressed genes were primarily enriched in the cell signaling pathways. The protein-protein interaction (PPI) network indicated that the hub differentially co-expressed genes of STIL were CDK1, CCNB2, CDC20, CCNA2, BUB1, and AURKB.

**Conclusions:**

STIL is associated with osteosarcoma proliferation and invasion, and may be promote the progression of osteosarcoma by regulating the expression of CDK1, CCNB2, CDC20, CCNA2, BUB1 and AURKB.

## Background

Among children and adolescents, osteosarcoma represents one of the most common type of malignant bone tumor, with an incidence of approximately 3 in 1 million people each year [1]. Osteosarcoma originates from the mesenchymal tissue, which is mainly located in the metaphysis of the long bones and is the most common around the knee joint [2]. Since osteosarcoma has the characteristics of rapid cell proliferation, strong invasive ability, and early metastasis [3, 4], the patient 5-year survival rate with non-metastatic and metastatic osteosarcoma is still only 60 % and 30 %, respectively [5–7], despite advances in treatment techniques (e.g., surgery and chemotherapy). Therefore, there is an urgent need to gain in-depth insight into the molecular mechanisms that result in the development of osteosarcoma and identify novel molecular therapeutic targets.

STIL is an important factor in cellular mitosis and centriole replication. In the cell cycle, STIL expression increases gradually during the early stages, peak in the middle stage, and rapidly decreases in the later stage [8]. Throughout this process, STIL interacts with a variety of proteins to ensure mitotic stability [9–11]. Thus, changes in STIL expression are associated with chromosome instability and even cancer. Some studies have demonstrated that STIL is overexpressed in a variety of cancers. Erez et al. reported that the high STIL expression in lung cancer tissue is both a marker of cell proliferation, as well as a marker of the metastatic potential of lung cancer [12]. Moreover, the study by Ouyang et al. found that high STIL expression in nasopharyngeal carcinoma may down-regulate the expression of ITGA2, Smad2, and JAK1, as well as promote the proliferation and invasion of nasopharyngeal carcinoma cells [13]. Despite these findings, the clinical significance and biological function of STIL in osteosarcoma remains unknown. Of particular interest is whether STIL is also highly expressed in osteosarcoma, and whether it promotes the progression of osteosarcoma as observed in other cancers. Therefore, this study sought to elucidate the biological function of STIL in osteosarcoma and the underlying molecular mechanism.

**Fig. 1:**
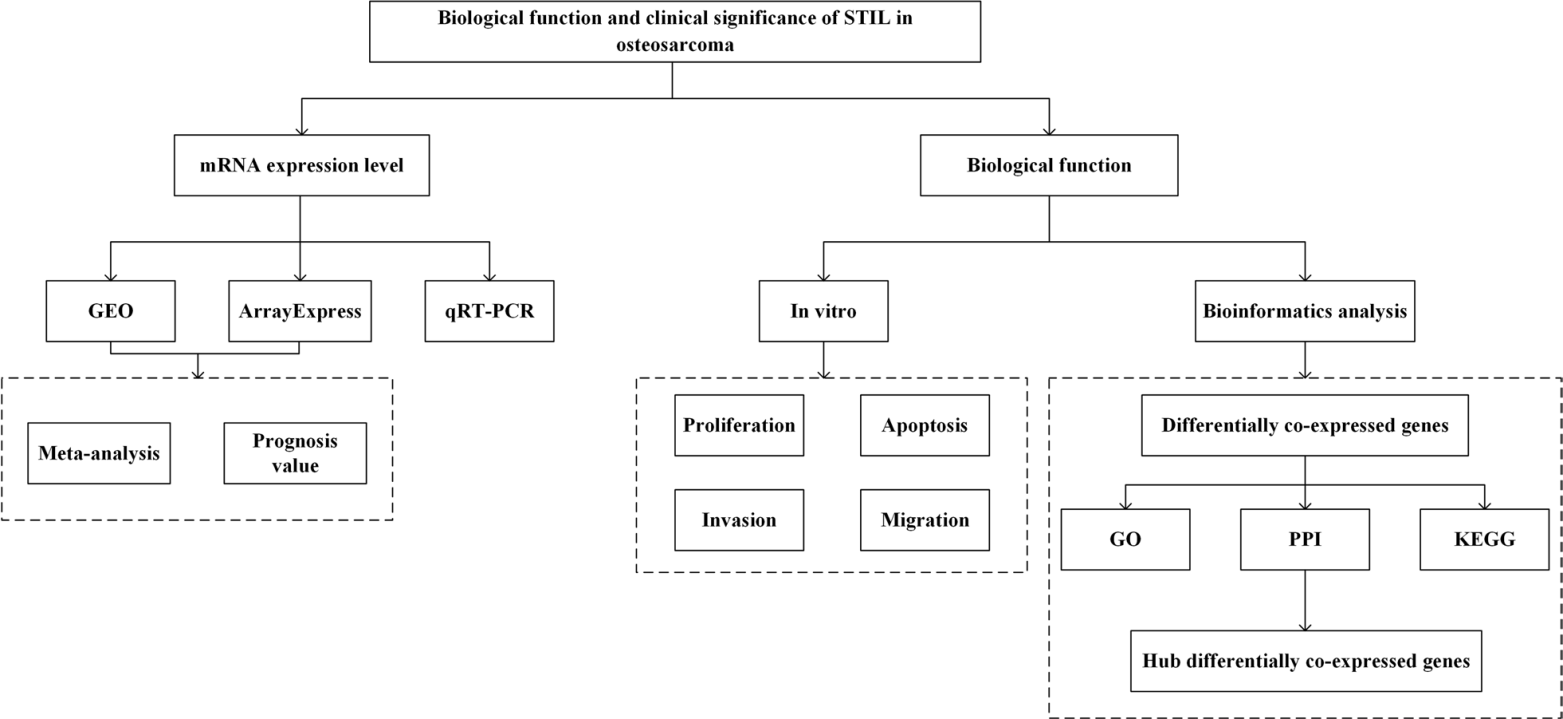
Flow diagram of the study

In this study, we first analyzed the clinical characteristics of STIL in osteosarcoma by integrating the gene chip data in public databases. Second, the biological functions of STIL in osteosarcoma were investigated using proliferation, apoptosis, migration and invasion experiments. Finally, we obtained the differentially co-expressed genes related to STIL, and the potential molecular mechanism was analyzed using bioinformatics methods. The flow chart of this study is shown in Fig. 1.

## Materials and methods

### Source of public databases

The gene microarray data were acquired from the ArrayExpress and Gene Expression Omnibus (GEO) databases, respectively. The following keywords were used to search the databases: “osteosarcoma” OR “osteosarcomas” AND “Homo sapiens”. The inclusion criteria of the data were as follows: (1) the experimental group and control group consisted of the osteosarcoma group and non-osteosarcoma group; (2) the patient did not receive radiotherapy, chemotherapy, or additional adjuvant therapy prior to the patient’s tissue was extracted; and (3) the number of samples in the control group and experimental group is ≥ 3. In addition, GSE21257 contains clinical data that can be used to analyze the clinical significance of STIL in osteosarcoma.

### Cell culture

Human osteosarcoma cells, U-2 OS, MG-63, and Saos2, as well as human osteoblast hFOB1.19 cells were obtained from the Cell Bank of the Chinese Academy of Sciences (Shanghai, China). Human osteosarcoma HOS cells were acquired from the Cell Bank of Wuhan University (Wuhan, China). MG-63 cells were cultured in Minimum Essential Medium (MEM, Gibco, USA), U-2 OS in McCoy’s 5A medium (Boster, Wuhan, China). Saos2 and HOS cells were cultured in Dulbecco’s modified Eagle’s medium (Gibco, USA) containing 1 % penicillin and streptomycin (Gibco, USA), 10 % fetal bovine serum (FBS, Gibco, USA) at 37℃. in a 5 % CO2 humidified incubator HFOB1.19 cells were maintained in MEM containing 10 % FBS, 1 % penicillin/streptomycin, and 0.3 mg/mL G418 (Gibco, USA) at 33.5 °C.

### Cell transfection

To down-regulate the expression of STIL in the two osteosarcoma cell lines (U-2 OS and HOS) exhibiting the highest STIL expression, three siRNA (A: GCAGGATGGTACCTTTCCA; B: CGAAGAGTATCCTATAAGA; C: GGAATACCAAATCAGTTAA) were synthesized from Guangzhou RiboBio Co., Ltd. (Guangzhou, China). According to the manual, Lipofectamine 2000 transfection reagent and Opti-MEM serum-lowering medium (Gibco, USA) was used to transfect the osteosarcoma cells.

### Quantitative reverse‐transcription polymerase chain reaction (qRT-PCR)

According to the manufacturer’s instructions, TRIzol (TAKARA, Japan) reagent was used to extract the total RNA. cDNA was synthesized using PrimeScript RT Master Mix (TAKARA, Japan). The cDNA combined with Power SYBR Green PCR Master Mix (Thermo Fisher, USA) were used for real-time PCR. The housekeeper gene, GAPDH, was used as a control. After normalizing GAPDH expression, the normal level of STIL expression in five cell lines was expressed as the relative expression and calculated in accordance with the 2-ΔΔCt method. The primer sequences in this study were as follows: GAPDH: 5′-TGACAACTTTGGTATCGTGGAAGG-3′ (F) and 5-AGGCAGGGATGATGTTCTGGAGAG-3′ (R). The following primers were used for STIL: 5′-CCCAACGCCAACTGGAGATTT-3′ (F) and 5-AGTCGGATGGTCTTCTCAGTC-3′ (R). Each experiment was repeated three times.

### Western blot (WB) analysis

The cell lysates were prepared using RIPA cleavage buffer and phenylmethanesulfonylfluoride fluoride (Beyotime Biotechnology, China), to extract the proteins from the cells after full cleavage. The protein was fractionated by SDS-polyacrylamide gel electrophoresis, electroblotted on 0.45 μm polyvinylidene difluoride membranes (Millipore, USA), blocked with 5 % skimmed milk powder at 37 °C for 1 h, washed three times with 1 × PBS-T (1000 mL 1×PBS + 0.5 mL Tween-20), and incubated with the primary antibody (diluted 1:1000) overnight at 4 °C. After three washes, a secondary antibody (diluted 1:10,000) was added to the membrane and incubated for 2 h, and the membrane was washed five times. Finally, an ECL kit (Beyotime Biotechnology, China) was used for detection. Anti-STIL and anti-GAPDH antibodies were purchased from Proteintech. A goat anti-mouse IgG (H + L) -HRP secondary antibody was purchased from Jackson ImmunoResearch. Each experiment was repeated three times.

### Proliferation assay

A Cell Counting Assay Kit-8 (CCK-8, Dojindo Laboratories, Japan) was used to analyze the transfected U-2 OS and HOS cells. Each type of cell was transferred into 96-well plates at 5000 cells/well, and CCK8 was added to each well of the plate and incubated for 24 h, 48 h, or 72 h, respectively. The optical density was measured using an enzyme labeling instrument at a wavelength of 450 nm. Each experiment was repeated three times.

### Cell apoptosis assay

Apoptotic cells were stained using a commercial FITC Annexin V Apoptosis Detection Kit I (Becton, Dickinson and Company, USA). The collected cells were washed with PBS, and resuspended in 100 μl Binding Buffer. The control group was resuspended in 400 μl Binding Buffer. Of the solution, 100 μl was mixed with 5 μl FITC-Annexin V and 5 μl PI, incubated for 15 min in the dark at 25 °C, and detected with a flow cytometer (Becton, Dickinson and Company, USA). Each experiment was repeated three times.

### Transwell migration and invasion assays

The invasion and migratory ability of the cells was evaluated using the Transwell method. To determine the migratory ability, the transfected osteosarcoma cells were suspended in serum-free medium (cell density: 2 × 10^5^/mL) and added to a Transwell chamber (8.0 μm; Becton, Dickinson and Company, USA). Next, 500 μl of complete medium was added to the lower chamber, and the plate was incubated for 48 h, fixed in paraformaldehyde, and washed with a crystal violet staining solution at room temperature for 20 min. The unbound crystal violet was washed away and the number of cells that had migrated were counted. The invasion ability involved the Transwell chamber to be covered with Matrigel (Becton, Dickinson and Company, USA) followed by steps similar to that used to assess the cell migration ability. Each experiment was repeated three times.

### Acquisition of STIL differentially co‐expressed genes in osteosarcoma

The DEGs in each gene chip was calculated using the Limma software package, with LogFC ≥ 1 and p < 0.05 as the standards. Genes that appeared in at least two studies were selected as DEGs. In addition, the correlation between STIL and other genes in each gene chip was assessed. STIL co-expressed genes were selected using the Pearson’s correlation coefficient r ≥ 0.5 and p < 0.05 as the screening criteria, as well as genes that appeared in at least two studies. The genes obtained from the intersection of DEGs and co-expressed genes were considered to be STIL differentially co-expressed genes in osteosarcoma.

### Enrichment analysis of STIL differentially co‐expressed genes and PPI analysis

The network tool, David (https://david.ncifcrf.gov/), was used to perform an enrichment analysis of the GO and KEGG pathways for the obtained differentially co-expressed genes. In addition, the differentially co-expressed genes were used to construct a PPI network through the STRING (https://string-db.org/) database, and Cytoscape 3.8.0 was used to visualize the PPI network with a score of 0.9 or higher.

### Statistical analysis

SPSS23.0 statistical software was used to perform an independent sample *t*-test, and the results were displayed as the mean ± standard deviation. SMD and summarized receiver operating characteristic (SROC) were calculated using STIL expression by STATA15.0. The Q test of the χ^2^ test and I^2^ were used to detect heterogeneity. When p > 0.05 and I^2^ < 50 %, there was considered to be no heterogeneity, and the use of a fixed effects model was employed. Alternatively, a continuous variable meta-analysis was performed in conjunction with a random effects model. The overall results were subjected to a sensitivity analysis to determine the effects of each gene chip. Publication bias was evaluated using Begg’s and Egger’s tests. In addition, the Kaplan-Meier method was used for survival analysis, and the difference in the survival rate was calculated with a log-rank test. Statistical significance was considered at p < 0.05.

## Results

### STIL expression in osteosarcoma

**Fig. 2:**
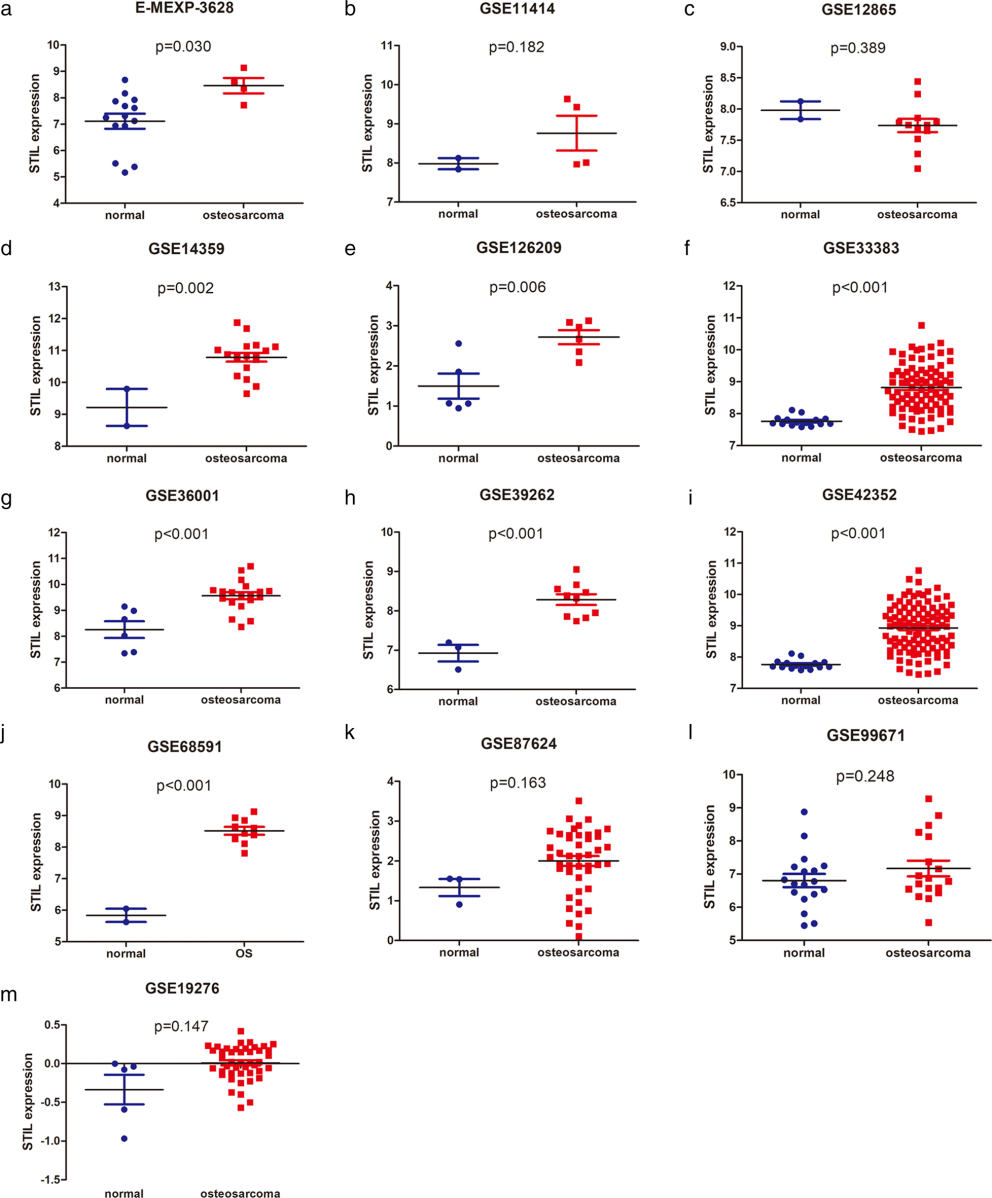
STIL scatter plot based on osteosarcoma chips in the GEO and ArrayExpress databases. **a** E-MEXP-3628, **b** GSE11414, **c** GSE12865, **d** GSE14359, **e** GSE126209, **f** GSE33383, **g** GSE36001, **h** GSE39262, **i** GSE42352, **j** GSE68591, **k** GSE87625, **l** GSE99671, and **m** GSE19276. STIL, SCL/TAL1 interrupting locus. *GEO* Gene Expression Omnibus

Based on the inclusion and exclusion criteria, we screened 13 osteosarcoma chips (Table 1). The STIL expression data were extracted from the 13 gene chips. As shown in Fig. 2, STIL was highly expressed in osteosarcoma tissues compared to normal tissues in the E-MEXP-3628, GSE14359, GSE126209, GSE33383, GSE36001, GSE39262, GSE42352, and GSE68591 chips. No highlighted difference was found between osteosarcoma tissues and normal tissues in the GSE11414, GSE12865, GSE87624, GSE99671, and GSE19276 chips.

**Table 1:** Expression of STIL based on GEO Database and ArrayExpress Database

Datasets	Country	Year	Platform	Tumor			Normal			t-value	p-value
				Number	Mean	SD	Number	Mean	SD		
GSE12865	Canada	2008	GPL6244	12	7.7365	0.36988	2	7.9813	0.2015	0.893	0.389
GSE19276	Australia	2009	GPL6848	44	0.0091	0.21813	5	− 0.3351	0.42831	− 1.771	0.147
GSE14359	Germany	2009	GPL96	18	10.7865	0.57156	2	9.2184	0.81967	− 3.577	0.002
GSE11414	Canada	2008	GPL6244	4	8.7616	0.89605	2	7.9813	0.2015	− 1.66	0.182
GSE36001	Norway	2012	GPL6102	19	9.5635	0.59652	6	8.2566	0.79049	− 4.336	< 0.001
GSE42352	Norway	2012	GPL10295	103	8.9301	0.74961	15	7.7656	0.15193	− 13.924	< 0.001
GSE39262	United Kingdom	2012	GPL96	10	8.2847	0.42951	3	6.9248	0.36673	− 4.933	< 0.001
GSE68591	USA	2015	GPL11028	10	8.5187	0.39898	2	5.8393	0.29784	− 8.868	< 0.001
GSE99671	Estonia	2017	GPL20148	18	7.1706	1.00465	18	6.806	0.85005	− 1.175	0.248
GSE126209	China	2019	GPL20301	6	2.7168	0.42522	5	1.497	0.69451	− 3.59	0.006
GSE87624	USA	2016	GPL11154	44	2.001	0.80613	3	1.3309	0.37029	− 1.418	0.163
GSE33383	Norway	2011	GPL10295	84	8.8196	0.74379	15	7.7656	0.15193	− 11.693	< 0.001
E-MEXP-3628		2012		4	8.4594	0.58655	14	7.1105	1.06811	− 2.389	0.030
GSE21257	Norway	2010	GPL10295	53	9.2958	0.806867					

**Fig. 3:**
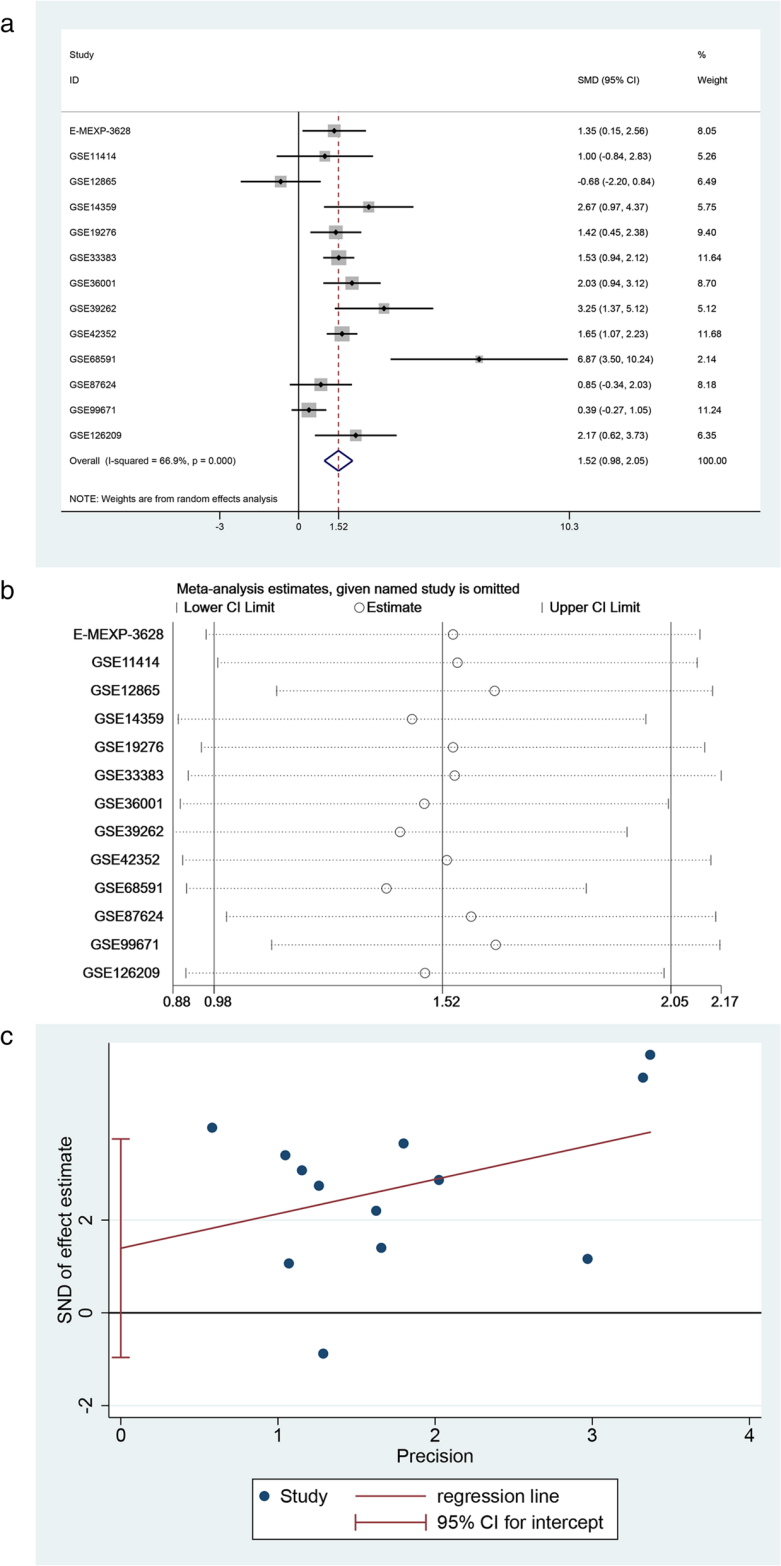
Evaluation of the level of STIL expression. **a **The level of STIL expression in osteosarcoma expressed as a forest plot based on a random effects model. **b **Sensitivity analysis of STIL expression in osteosarcoma. **c** Egger’s test to detect publication bias

In addition, in the meta-analysis of 13 chips, p < 0.05, and an I^2^ value of 66.9 % were considered to be heterogeneous. Therefore, the random effects model was used to conduct a variable continuous meta-analysis. STIL expression in 376 osteosarcoma samples was higher than that in 92 normal samples, and the total SMD integrated by the random effects model was 1.52 (95 % confidence interval [CI] 0.98–2.05) (Fig. 3a). The sensitivity analysis suggested that the exclusion of any chip data had little effect on the total effect (Fig. 3b). The results of the Begg’s test showed that p = 0.180 had no publication bias, while the Egger’s test showed an absence of publication bias (Fig. 3c).

### The clinical diagnostic ability of STIL

**Fig. 4:**
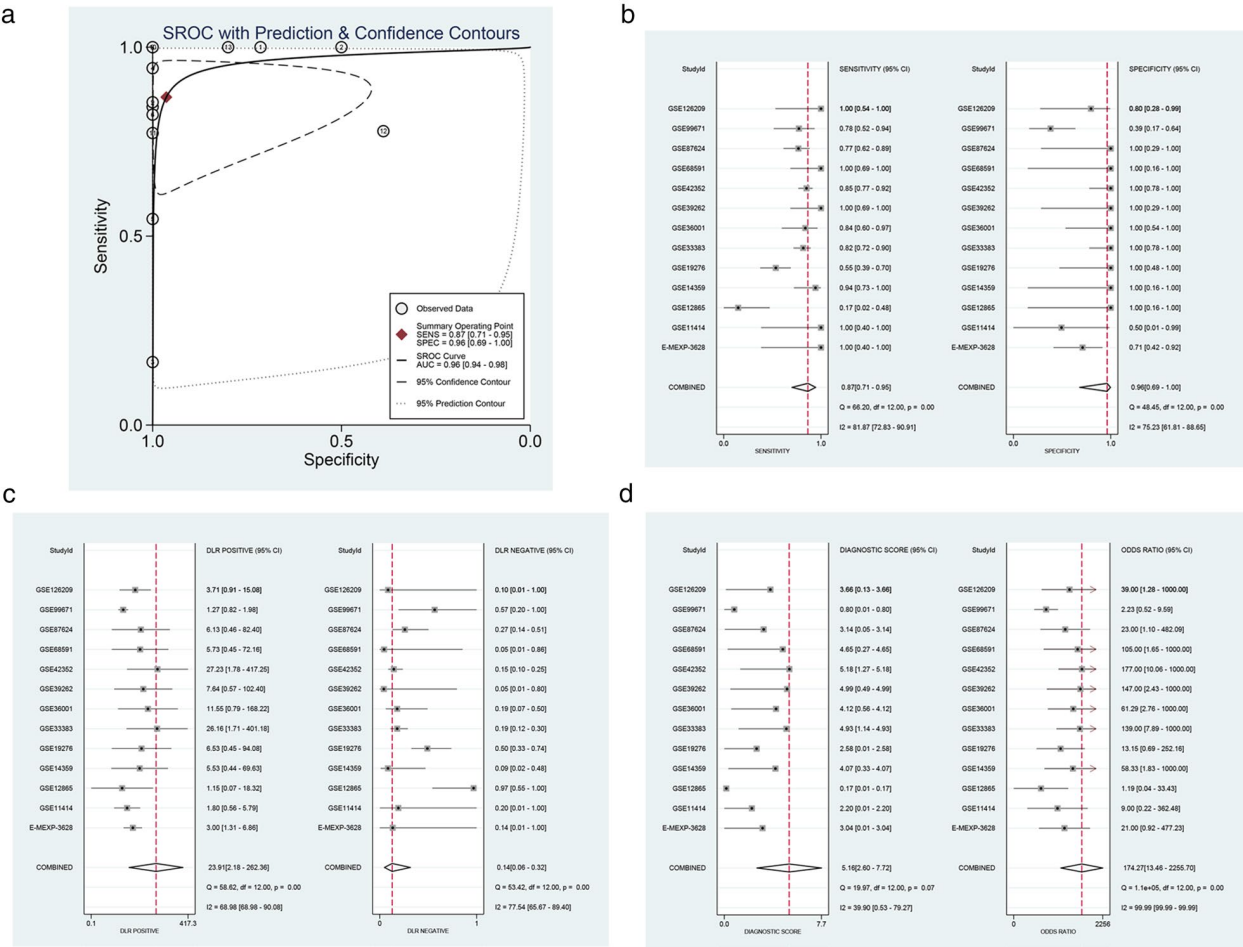
The ability of STIL to differentiate between osteosarcoma tissue and normal tissue. **a** SROC curve of STIL and **b** combined sensitivity and specificity of STIL. **c** The combined positive and negative likelihood ratio of STIL. **d** The combined diagnostic score and diagnostic odds ratio of STIL. *SROC* summarized receiver operating characteristic

To explore the diagnostic ability of STIL for osteosarcoma, we used the SROC curve and related analysis. The results of the SROC curve showed that the AUC was 0.96 (95 % CI 0.94–0.98), and STIL can distinguish osteosarcoma from non-osteosarcoma (Fig. 4a). The combined sensitivity and specificity were 0.89 (95 % CI 0.75–0.95) and 0.98 (95 % CI 0.68–1.00), respectively (Fig. 4b). The combined positive likelihood ratio and negative likelihood ratio were 37.44 (95 % CI 2.22–630.61) and 0.11 (95 % CI 0.05–0.26) (Fig. 4c). The diagnostic score and diagnostic odds ratio were 5.79 (95 % CI 3.18–8.39) and 325.99 (95 % CI 24.14–4401.95), respectively (Fig. 4d). The above results indicate that STIL has good clinical diagnostic ability.

### Effect of STIL on osteosarcoma cell proliferation and apoptosis

**Fig. 5:**
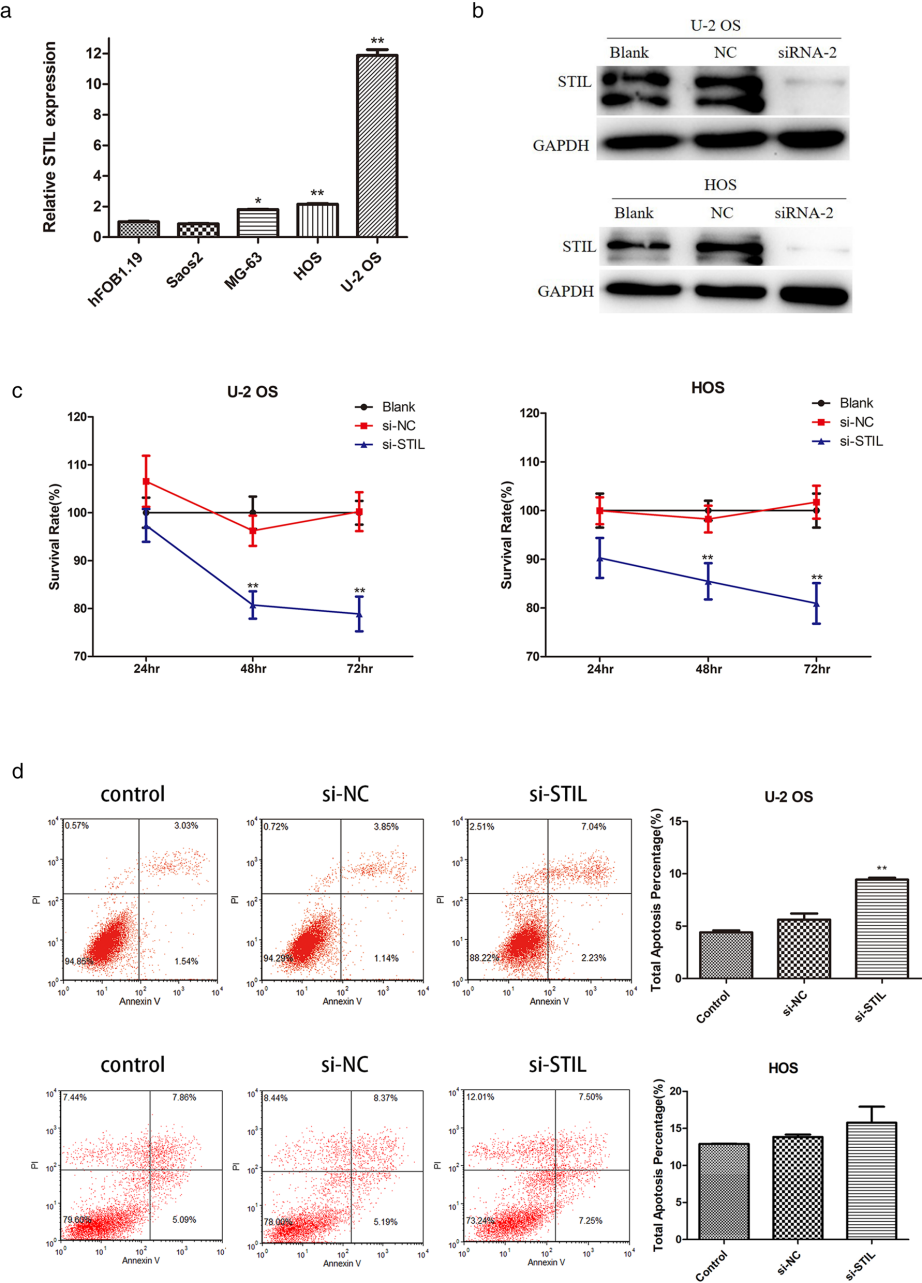
Effect of silencing STIL on the proliferation and apoptosis of osteosarcoma cells. **a** qRT-PCR detection of STIL expression in osteoblast and osteosarcoma cell lines. **b** WB detects the silencing efficiency of STIL. **c** CCK8 analysis showing that silencing STIL inhibited the proliferation of U-2 OS and HOS cell lines. **d** Apoptosis tests showed that silencing STIL promoted the apoptosis of the U-2 OS cell line. *p < 0.05; **p < 0.01 vs. hFOB1.19 or si-NC

To study the expression of STIL in osteosarcoma cells, we detected the level of STIL mRNA by qRT-PCR. Of the osteoblast cell lines and four osteosarcoma cell lines, it was found that the up-regulation of STIL mRNA was the most significant in the U-2 OS and HOS cell lines (Fig. 5a). In addition, to study the effect of STIL on osteosarcoma cells, we inhibited STIL expression in the two cell lines with the highest expression levels (U-2 OS and HOS). The WB results showed that the knockdown effect at the siRNA-2 site was the most significant (Fig. 5b). A CCK8 assay was used to observe the effect of STIL on osteosarcoma cell proliferation, and the results revealed that a knockdown of STIL could significantly inhibit the proliferation of osteosarcoma cells (Fig. 5c). In addition, the apoptosis experiments revealed that compared with the control group, there was an increase in the level of apoptotic osteosarcoma cells after knocking down STIL, which was statistically significant in U-2 OS cells, but not in HOS cells (Fig. 5d). These results indicate that silencing STIL inhibits proliferation and increases apoptosis in osteosarcoma cells.

### Effect of STIL on osteosarcoma cell migration and invasion

**Fig. 6:**
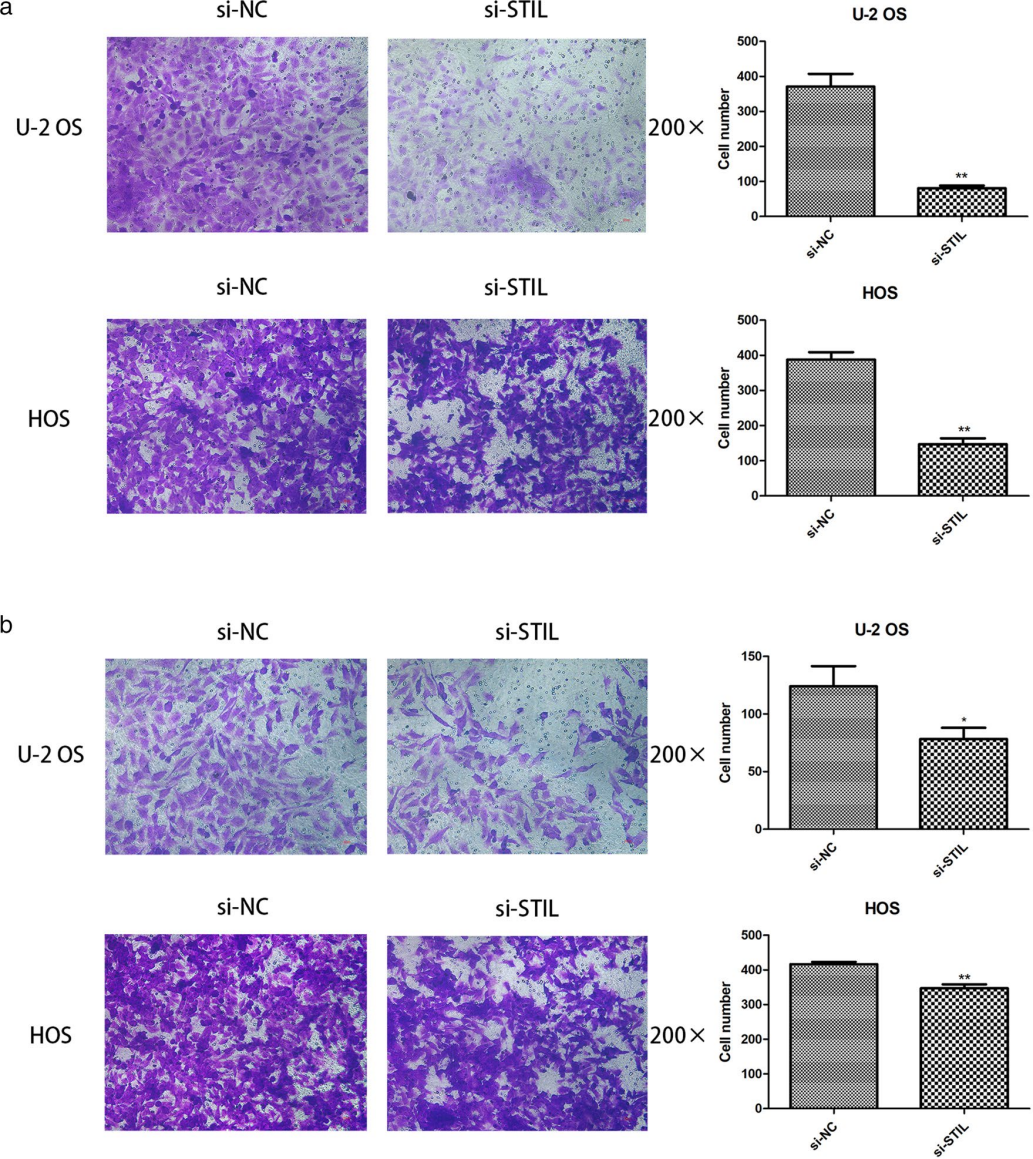
The effect of silencing STIL on the migration and invasion of osteosarcoma cells. **a** Transwell migration experiment showing that silencing STIL inhibits the migratory ability of osteosarcoma cell lines. **b** Transwell invasion experiment demonstrating the ability of silencing STIL to inhibit the invasion of osteosarcoma cell lines. *p < 0.05; **p < 0.01

Next, we conducted a Transwell assay to study the effect of STIL on the migration and invasion of osteosarcoma cells. The Transwell assay results revealed that osteosarcoma cell migration was significantly inhibited after STIL was silenced (Fig. 6a). In addition, the invasive ability was tested and there was a significantly weakened invasive ability of STIL compared to that of the control group after STIL was silenced in the two cell lines (Fig. 6b). These results indicate that silencing STIL inhibits the migration and invasion of osteosarcoma cells.

### Expression and clinical characteristics of STIL

**Fig. 7:**
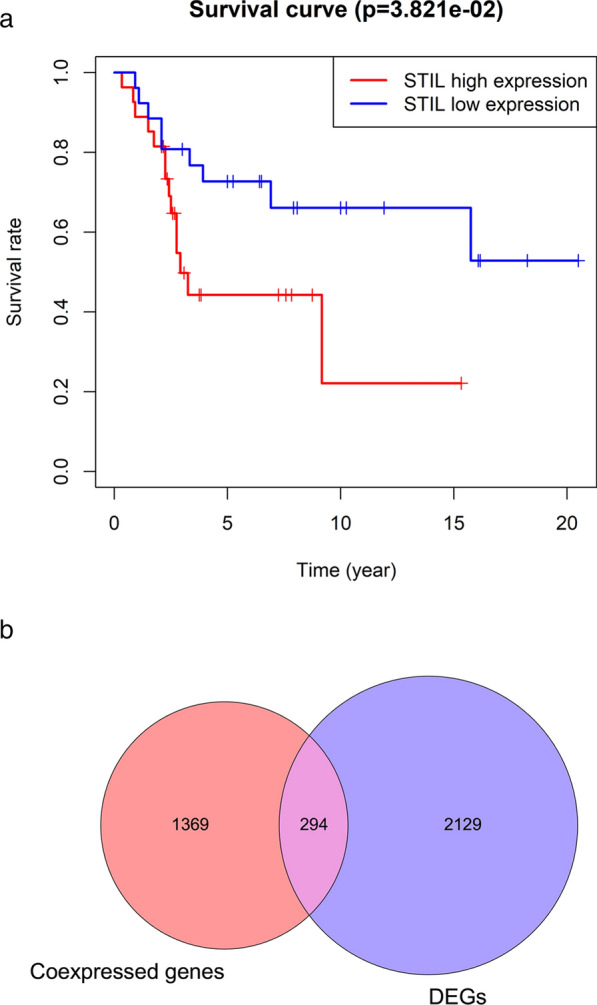
Survival analysis of STIL and differentially co-expressed genes. **a** Survival analysis of STIL based on the GSE21257 chip. **b** Venn diagram showing the STIL differentially co-expressed genes in osteosarcoma in the GEO and ArrayExpress databases. *GEO* Gene Expression Omnibus

To study the prognostic value of STIL in osteosarcoma, the data of 53 osteosarcoma cases were extracted from the GSE21257 tissue microarray and their clinical features were analyzed (Table 2). A significant difference was observed in the level of STIL expression between the group aged < 16 years old and that aged ≥ 16 years old (p < 0.010). A significant difference was also observed between the metastasis and non-metastasis groups (p < 0.007), whereas there was no statistical significance based on gender. At the same time, the survival analysis in Fig. 7a shows that the prognosis of high STIL expression was lower than that of low STIL expression, and the 5-year survival rates were 0.44 and 0.727, respectively (p < 0.05). The above results indicate that STIL may have prognostic value.

**Table 2:** Relationship between STIL expression and clinical parameters in GSE21257 microarray

Clinical parameters	Group	STIL expression		t‑value	p‑value
		Cases	Mean ± SD		
Tissue	Osteosarcoma tissue	53	9.2958 ± 0.806867		
Age(years)	< 16	23	9.6153 ± 0.81223	2.668	0.010
	≥ 16	30	9.0508 ± 0.72380		
Sex	Male	34	9.4181 ± 0.84153	− 1.494	0.141
	Female	19	9.0768 ± 0.70963		
Metastasis	Yes	34	9.5203 ± 0.74956	− 2.88	0.007
	No	19	8.8939 ± 0.76487		

*STIL* SCL/TAL1 interrupting locus, *SD* standard deviation

### GO and KEGG analysis of STIL differentially co‐expressed genes

**Fig. 8:**
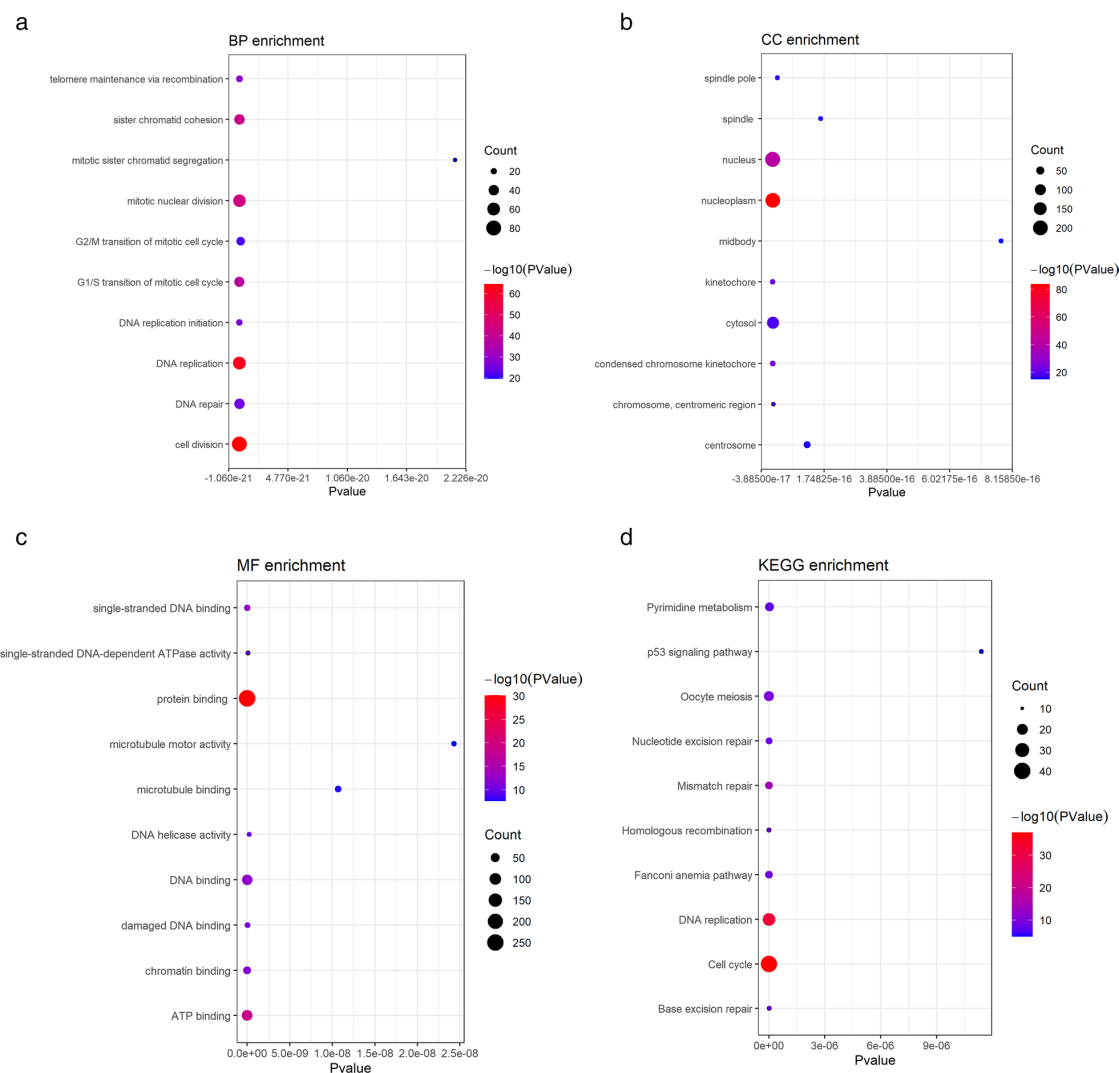
GO and KEGG analysis of 294 STIL differentially co-expressed genes. **a** Bubble chart of the first 10 Biological processes. **b** Bubble chart of the first 10 cellular components. **c** Bubble chart of the first 10 molecular functions. **d** The first 10 KEGG pathways. *GO* Gene Ontology, *KEGG* Kyoto Encyclopedia of Genes and Genomes

A total of 2425 DEGs and 1663 STIL co-expressed genes were screened from the public database, and there were 294 differentially co-expressed genes identified in the overlapping regions (Fig. 7b). The GO enrichment analysis of STIL differentially co-expressed genes (Fig. 8a–c) revealed that the biological process (BP) was primarily enriched in the areas of cellular and mitotic nuclear division, as well as sister chromatid cohesion. The nucleoplasm, kinetochore, and spindle exhibited the primary enrichment of cellular component (CC). Protein binding, ATP binding, and single-stranded DNA-dependent ATPase activity were mainly enriched in molecular function (MF). The KEGG analysis indicated that the primary enrichment of the STIL differentially co-expressed genes was in the areas of cell cycle, mismatch repair, and oocyte meiosis (Fig. 8d). Tables 3 and 4 list the top 10 most significant pathways in the GO and KEGG analyses.

**Table 3:** The 10 most important items of STIL differentially co-expression genes in the GO analysis

Category	GO ID	GO Term	Count	p‑value
BP	GO:0051301	Cell division	33	1.63E−17
BP	GO:0007067	Mitotic nuclear division	26	7.98E−15
BP	GO:0007062	Sister chromatid cohesion	18	5.42E−14
BP	GO:0006281	DNA repair	21	9.69E−11
BP	GO:0006260	DNA replication	16	3.98E−09
BP	GO:0007080	Mitotic metaphase plate congression	9	3.22E−08
BP	GO:0000070	Mitotic sister chromatid segregation	8	3.41E−08
BP	GO:0051726	Regulation of cell cycle	13	1.48E−07
BP	GO:0008283	Cell proliferation	20	8.32E−07
BP	GO:0000732	Strand displacement	7	1.19E−06
CC	GO:0005654	Nucleoplasm	98	1.45E−20
CC	GO:0000776	Kinetochore	15	3.76E−12
CC	GO:0005819	Spindle	17	7.57E−12
CC	GO:0030496	Midbody	17	2.05E−11
CC	GO:0005829	Cytosol	89	3.50E−11
CC	GO:0000922	Spindle pole	13	2.76E−08
CC	GO:0000775	Chromosome, centromeric region	10	6.58E−08
CC	GO:0005876	Spindle microtubule	9	1.15E−07
CC	GO:0000777	Condensed chromosome kinetochore	11	2.69E−07
CC	GO:0005737	Cytoplasm	105	2.99E−06
MF	GO:0005515	Protein binding	184	2.28E−17
MF	GO:0005524	ATP binding	48	6.44E−08
MF	GO:0043142	Single-stranded DNA-dependent ATPase activity	6	1.14E−07
MF	GO:0019901	Protein kinase binding	21	2.85E−07
MF	GO:0000400	Four-way junction DNA binding	5	3.18E−05
MF	GO:0003697	Single-stranded DNA binding	9	4.34E−05
MF	GO:0019899	Enzyme binding	16	6.31E−05
MF	GO:0003690	Double-stranded DNA binding	8	1.27E−04
MF	GO:0003689	DNA clamp loader activity	4	1.38E−04
MF	GO:0008022	Protein C-terminus binding	11	2.23E−04

*GO* gene ontology, *BP* biological process, *CC* cellular component, *MF* molecular function

**Table 4:** 10 KEGG pathways most related to STIL and co-expressed genes

Category	KEGG ID	KEGG term	Count	p-value
KEGG	hsa04110	Cell cycle	16	2.82E−09
KEGG	hsa03430	Mismatch repair	6	3.89E−05
KEGG	hsa04114	Oocyte meiosis	10	1.26E−04
KEGG	hsa03440	Homologous recombination	6	1.26E−04
KEGG	hsa03030	DNA replication	5	3.37E−03
KEGG	hsa04914	Progesterone-mediated oocyte maturation	7	3.98E−03
KEGG	hsa05212	Pancreatic cancer	6	5.33E−03
KEGG	hsa05166	HTLV-I infection	11	1.30E−02
KEGG	hsa04115	p53 signaling pathway	5	2.91E−02
KEGG	hsa03010	Ribosome	7	3.12E−02

*KEGG* Kyoto Encyclopedia of Genes and Genomes

### PPI network analysis

**Fig. 9:**
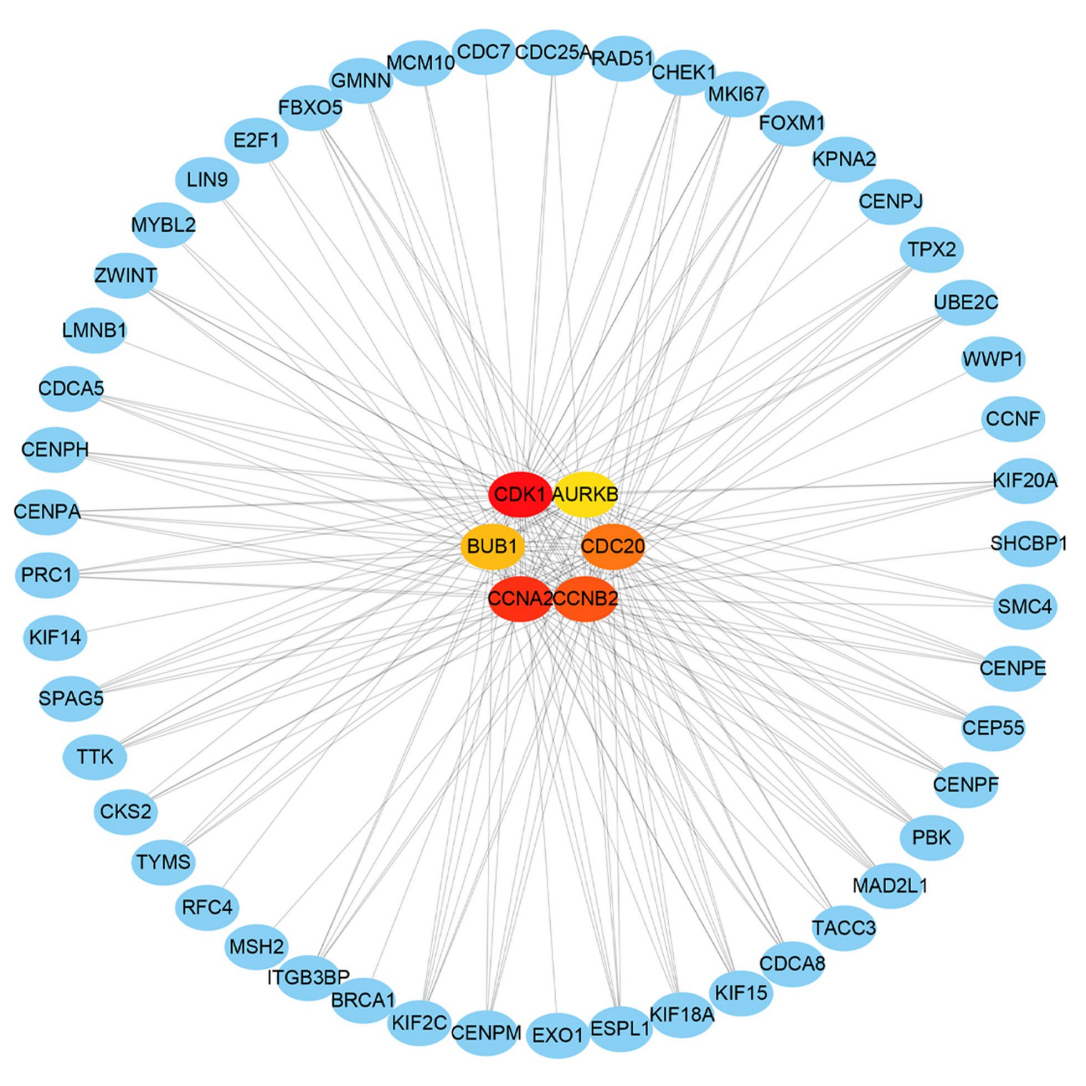
PPI analysis of the STIL differentially co-expressed genes in osteosarcoma. Six Hub genes were selected in the PPI network of STIL differentially co-expressed genes. *PPI* protein-protein interaction

To identify the differentially co-expressed genes closely related to STIL, the STIL differentially co-expressed genes were analyzed by PPI network. The first six genes (CDK1, CCNB2, CDC20, CCNA2, BUB1, and AURKB) were selected as hub differentially co-expressed genes by degree analysis in Cytoscape (Fig. 9). Therefore, these six hub differentially co-expressed genes were considered to be the key genes that interact with STIL.

### Validation of hub differentially co‐expressed genes based on 13 gene chips

**Fig. 10:**
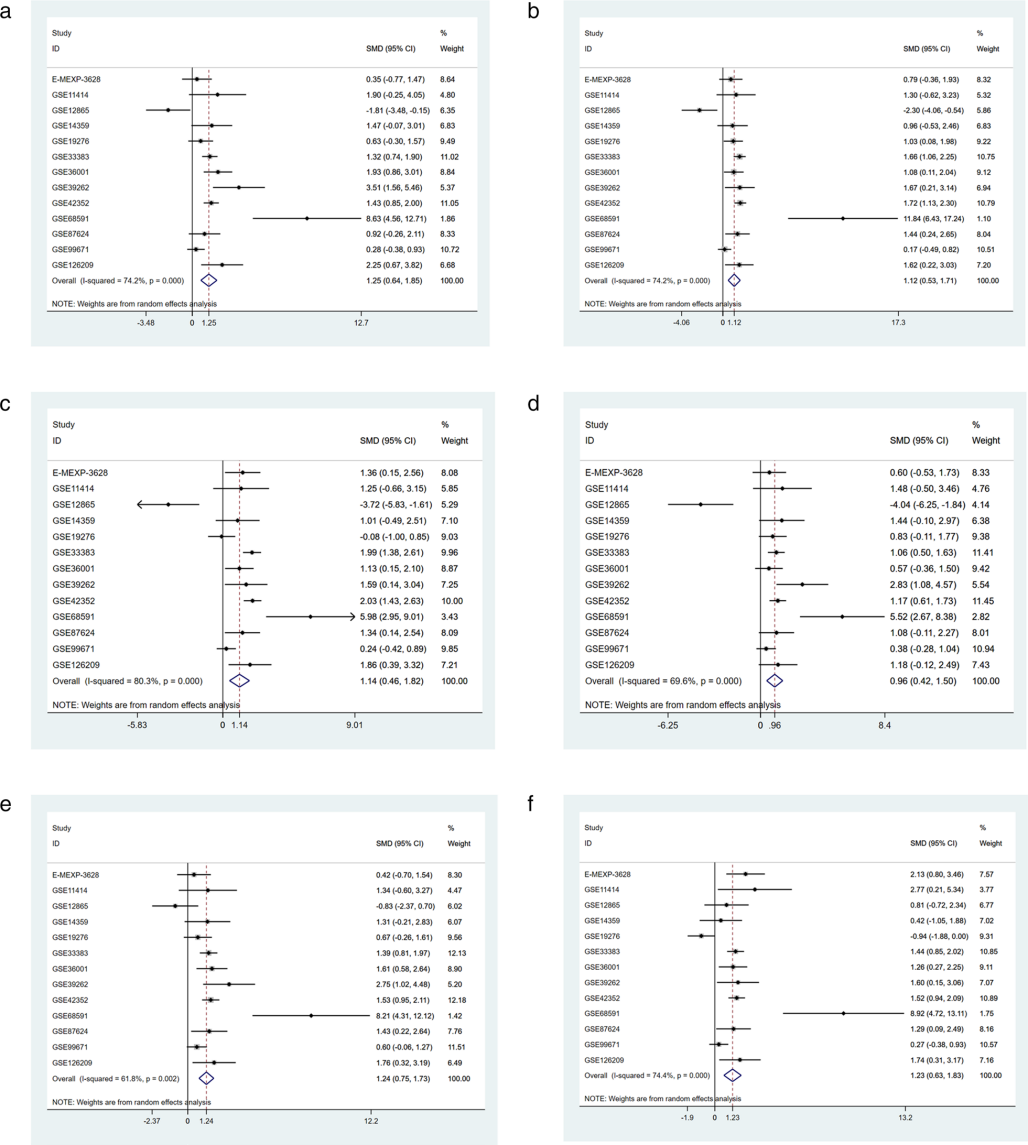
Evaluation of levels of Hub gene expression in the GEO and ArrayExpress databases. **a** CDK1, **b** CCNB2, **c** CDC20, **d** CCNA2, **e** BUB1, and **f** AURKB. *GEO* Gene Expression Omnibus, *GEO* Gene Expression Omnibus

**Fig. 11:**
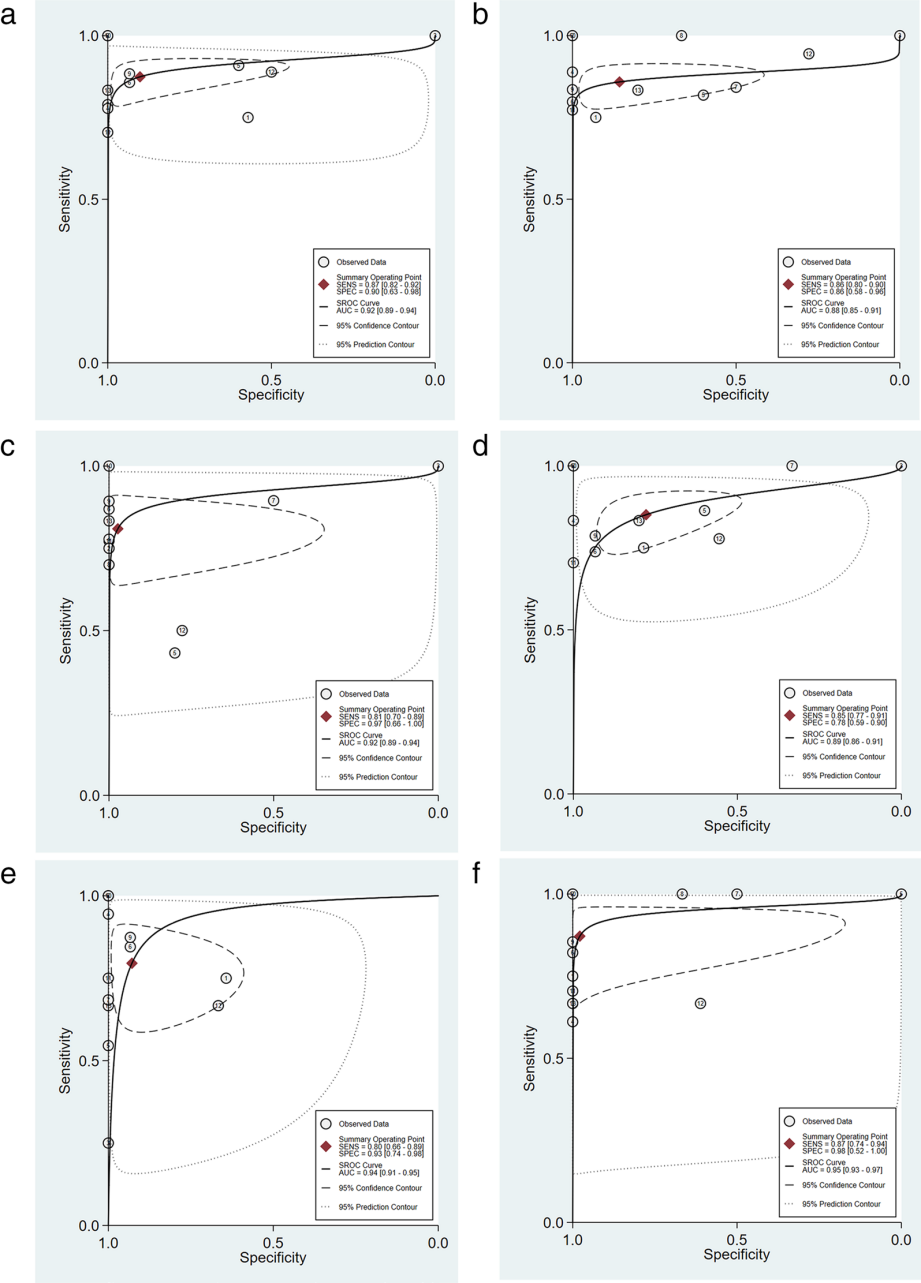
SROC curve based on Hub gene expression in the GEO and ArrayExpress databases. **a** CDK1, **b** CCNB2, **c** CDC20, **d** CCNA2, **e** BUB1, and **f** AURKB. *GEO* Gene Expression Omnibus, *SROC* summarized receiver operating characteristic

Using the 13 gene chips, we found that six hub differentially co-expressed genes (CDK1, CCNB2, CDC20, CCNA2, BUB1, and AURKB) were highly expressed in osteosarcoma (Fig. 10). In addition, according to the SROS curve results, these genes also exhibit the ability to distinguish osteosarcoma from non-osteosarcoma samples (Fig. 11).

## Discussion

In this study, 13 gene chips were collected and it was found that STIL expression was significantly increased in osteosarcoma. Highly expressed STIL was associated with the ability to distinguish osteosarcoma from non-osteosarcoma samples, and the patients with high STIL expression are associated with a poor prognosis. Additionally, the in vitro experiments revealed that the silencing of STIL could significantly block proliferation, induce apoptosis, and reduce migration and invasion in osteosarcoma cells. Previous research has identified a wide range of molecular markers that can be used for the diagnosis and treatment of various diseases [14–16]. Moreover, several molecular markers for osteosarcoma have also been discovered, including MYC, Cyclin E1, and MiR-455-3p, which are all considered to be promising therapeutic targets [17–19]. In this study, STIL was found to play a role of a proto-oncogene in osteosarcoma and was significantly involved in the occurrence and development of osteosarcoma. Compared with previous studies, our findings provide new insight into molecular markers and therapeutic targets for osteosarcoma.

STIL was first isolated from T cell chromosomes from T cell acute lymphoblastic leukemia [20]. Early studies that commonly used mice and zebrafish as animal models found that a deletion of the STIL locus was embryonically lethal [21–23]. Moreover, STIL mutations in humans can lead to primary hereditary microcephaly and even cancer [24–26]. This is the first study to report the overexpression of STIL in osteosarcoma, and demonstrate that silencing STIL inhibits cell proliferation, promotes cell apoptosis, and suppresses invasion and migration capabilities. Kasai et al. found that STIL was able to regulate the Hh signaling pathway through interacting with Sufu and Gli1 to affect cell proliferation [11]. Wu et al. found STIL to be highly expressed in prostate cancer and could regulate the growth of prostate cancer cells through the MAPK/ERK, PI3K/Akt, and AMPK signaling pathways [27]. Furthermore, Rabinowicz et al. reported that the targeted inhibition of highly expressed STIL could significantly improve the efficacy of DNA-damaging drugs for the treatment of ovarian cancer, and suggested that STIL might be a novel therapeutic target [28]. In recent studies, Wang et al. attenuated the IGF-1/PI3K/AKT pathway by knocking out STIL in gastric cancer, which inhibited cellular proliferation and reduced clone formation ability [29]. These results are consistent with our results and provide evidence for the role of STIL as a proto-oncogene.

To further clarify the potential molecular mechanism of STIL in osteosarcoma, we performed a GO and KEGG enrichment analysis on the STIL differentially co-expressed genes, and they were found to be significantly enriched in the cell cycle pathway. The PPI network results also showed that STIL and the hub co-expressed genes were proteins involved in the cell cycle. The above results indicate that STIL and the differentially co-expressed genes may affect the mitosis and cell proliferation of osteosarcoma cells through cell cycle signaling pathways. The cell cycle is known to have an important function in cellular growth and proliferation. Prior studies have reported that miR-671-5p and miR-299-5p target cell cycle regulation and mediate osteosarcoma proliferation [30, 31]. Zhang et al. found that Ludartin induces apoptosis and cell cycle arrest at the G2/M checkpoint through the elevated expression of p21WAF1 in osteosarcoma cells [32]. Cell cycle pathways have also been established to play an important role in other tumors [33–35]. As an important factor in the process of mitosis, STIL may participate in the progression of osteosarcoma by regulating the cell cycle.

In the PPI network, we selected six genes (CDK1, CCNB2, CDC20, CCNA2, BUB1, and AURKB) as the core STIL differentially co-expressed genes in osteosarcoma. CDK1 is a gene related to the cell cycle, and its abnormal expression leads to the development of tumors [36]. In the study conducted by Huang et al., microRNA-199a-3p, as a tumor suppressor gene was found to exhibit low expression in osteosarcoma and may interact with highly expressed CDK1 in the development of osteosarcoma [37]. CCNA2 and CCNB2 are members of cyclin family, which are critical for both cellular proliferation and apoptosis. Shekhar et al. found that CCNA2 is the common target of miR-449a and miR-424 in osteosarcoma, which inhibits tumor progression by inhibiting CCNA2 expression [38]. In another study, silencing CDC6 reduced CCNA2 expression and suppressed osteosarcoma cell proliferation and invasion [39]. CDC20 is a gene that regulates the cell cycle, and is reported to be involved in osteosarcoma development by analyzing the gene chip data [40]. Moreover, apcin blocks osteosarcoma cell growth and invasion by reducing the level of CDC20 expression, indicating that CDC20 may represent a potential therapeutic target [41, 42]. In a recent study, CDC20 was found to exhibit high levels of expression in osteosarcoma cisplatin-resistant cell lines, which enhanced the sensitivity of drug-resistant cell lines to cisplatin by knocking out CDC20 [43]. Moreover, studies using bioinformatics analyses have found that RFC4 may interact with BUB1, which may function to promote osteosarcoma occurrence and development of [44]. AURKB is a serine/threonine kinase that has been proposed to stimulate the invasion and proliferation of osteosarcoma through PTK2/PI3K/AKt/NF-κB signaling pathway and VCP [45, 46]. Thus, AURKB inhibitors may also provide a new option for the treatment of osteosarcoma [47]. In our study, we found that these six genes are highly expressed in osteosarcoma. Therefore, these findings suggest that STIL may play a role in osteosarcoma progression through regulation of expression of these identified genes.

Our study is associated with certain limitations: (1) there is a large heterogeneity in our analysis (67 %). Although we attempted to identify the source of heterogeneity through a sensitivity analysis, the results showed that no particular research to be the source of the heterogeneity; and (2) although we have demonstrated that STIL functions as a proto-oncogene in osteosarcoma, its potential molecular mechanism requires further verification both in vivo and in vitro.

## Conclusions

In general, we confirmed that STIL plays a role as a proto-oncogene, is highly expressed in osteosarcoma, and is involved in osteosarcoma proliferation and invasion. Moreover, the bioinformatics analysis showed that STIL may participate in the biological function of osteosarcoma by regulating CDK1, CCNB2, CDC20, CCNA2, BUB1, and AURKB. These findings provide a new perspective for the use of STIL as a potential biomarker and molecular therapeutic target for osteosarcoma.

## Data Availability

The datasets used and/or analyzed during the current study are available from the corresponding author on reasonable request.

## Abbreviations

STIL: SCL/TAL1 interrupting locus
DEGs: Differentially expressed genes
GO: Gene ontology
KEGG: Kyoto Encyclopedia of Genes and Genomes
SMD: Standardized mean difference
AUC: Area under the curve
PPI: Protein-protein interaction
GEO: Gene expression omnibus
MEM: Minimum essential medium
qRT-PCR: Quantitative reverse-transcription polymerase chain reaction
WB: Western blot
CCK-8: Cell Counting Assay Kit-8
SROC: Summarized receiver operating characteristic
CI: Confidence interval
BP: Biological process
CC: Cellular component
MF: Molecular function
